# Implementation and effectiveness of an intensive education program on phosphate control among hemodialysis patients: a non-randomized, single-arm, single-center trial

**DOI:** 10.1186/s12882-021-02441-8

**Published:** 2021-07-01

**Authors:** Jinmei Yin, Jun Yin, Rongli Lian, Peiqiu Li, Jing Zheng

**Affiliations:** 1 Blood purification center, The Fifth Affiliated Hospital of Sun Yat-sen University, Zhuhai, China; 2 Blood purification center, The Third Affiliated Hospital of Sun Yat-Sen University, Guangzhou, China; 3 Department of critical medicine, The Fifth Affiliated Hospital of Sun Yat-sen University, Zhuhai, China; 4 School of Nursing, Guangdong Pharmaceutical University, 283 Jianghai Avenue, Guangzhou, 510310 Guangdong Province China

**Keywords:** Hemodialysis, Phosphate, Medication adherence, Education

## Abstract

**Background:**

Hyperphosphatemia is a common complication in patients on maintenance hemodialysis. Patients’ adherence to phosphorus control can be improved by consistent education. However, few studies have focused on the model construction and effects of health education on phosphate control for hemodialysis patients.

**Objective:**

To develop an intensive education program focusing on phosphate control among hemodialysis patients and to analyze the effectiveness of this program.

**Design:**

A non-randomized, single-arm, single-center trial lasting for 6 months.

**Setting:**

This program was conducted in a hemodialysis center in a teaching hospital in Zhuhai, China.

**Participants:**

Patients on maintenance hemodialysis with hyperphosphatemia.

**Methods:**

An intensive hyperphosphatemia control education program lasting for 6 months was conducted among 366 hemodialysis patients applying the First Principles of Instruction model, which focused on mastering four stages: (a) activation of prior experience, (b) demonstration of skills, (c) application of skills and (d) integration of these skills into real-world activities. The controlled percentage of serum phosphorus, knowledge of hyperphosphatemia, and adherence to phosphate binders before and after the education program were assessed.

**Results:**

The proportion of controlled serum phosphorus was significantly increased from 43.5 to 54.9% (*P*<0.001). The scores on the knowledge of phosphate control were improved significantly from 59.0 ± 18.9 to 80.6 ± 12.4 (*P* < 0.001). The proportion of high adherence to phosphate binders was increased dramatically from 21.9 to 44.5% (*P* < 0.001).

**Conclusion:**

The intensive education program can effectively improve serum phosphorus, knowledge of hyperphosphatemia, and adherence to phosphate binders among hemodialysis patients.

**Trial registration:**

Chinese Clinical Trial Registry, ChiCTR2100042017. Retrospectively registered January 12th, 2021.

## Introduction

Maintenance hemodialysis is a widely used treatment for end-stage renal disease. In these patients, phosphate becomes easily retained in the body due to decreased renal clearance, leading to hyperphosphatemia, which is a common complication in these patients [1]. Hyperphosphatemia plays a key role in the development of secondary hyperparathyroidism, which can lead to mineral and bone disorder (MBD), metastatic calcification of heart valves, blood vessels, and soft tissues, and increased cardiovascular-related mortality [2, 3]. Hyperphosphatemia is an independent risk factor for mortality in chronic kidney disease (CKD) [4–6], which is also strongly associated with mortality in hemodialysis patients [7].

However, serum phosphorus control in hemodialysis patients is suboptimal. The Dialysis Outcomes and Practice Pattern Study (DOPPS) 5 in the U.S. showed that the prevalence of hyperphosphatemia in hemodialysis patients from 2012 to 2015 was 33%, while the one in China was 55% [8]. The Kidney Disease Outcomes Quality Initiative (KDOQI) clinical practice guidelines for bone metabolism and disease recommended that the target serum phosphorus level in patients with stage 5 CKD is ≤5.5 mg/dL [9]. According to this standard, the percentage of hemodialysis patients with a normal level of serum phosphorus was 49.8% [10] in 2007 and was improved slightly to 52.6% [11] in 2012 from DOPPS annual reports. A multicenter study on 1711 hemodialysis patients in 28 hospitals in China suggested that the corresponding percentage was considerably lower, at only 37.6% [12]. Thus, hyperphosphatemia control remains a great challenge for nephrology health care professionals in China.

## Background

The Kidney Disease: Improving Global Outcomes (KDIGO) foundation officially issued the guidelines, which recommended that serum phosphorus control could follow the “3D” treatment model: limiting dietary phosphate intake (diet), reducing phosphate levels with drugs (drugs), and undergoing hemodialysis (dialysis) [13]. In 2019, the newly issued CKD-Mineral and Bone Disorder guidelines in China [14] recommended that non-calcium-containing phosphate binders should be considered as first-line phosphate-lowering drugs, while the use of calcium-containing phosphate binders should be limited, and specialized intensive patient education should be used to improve serum phosphorus control.

Lacking knowledge about hyperphosphatemia and poor dietary adherence are the main reasons for poor serum phosphorus control, accounting for the increased mortality in hemodialysis patients with hyperphosphatemia [15]. Among hemodialysis patients, adherence to diet and medication for controlling hyperphosphatemia is not optimal. About 30% of the patients who need to take phosphate-binding agent do not pay attention to it [16]. Adherence to phosphate binders ranges from 22 to 71% (due to the different assessment methods used), with a mean of 47% [17]. Thus, scientific dietary and medication education for hemodialysis patients with hyperphosphatemia are the essential adjunct measures for hyperphosphatemia control. A meta-analysis [18] showed that intensive health education could significantly reduce serum phosphorus levels in maintenance hemodialysis patients.

An effective educational program for hemodialysis patients relies on the scientific design and appropriate teaching strategies used in the program and long-term and regular education. Merrill’s First Principles of Instruction (2002) is a new theory advocated by *M. David* Merrill, a well-known American educational researcher and educational psychologist. Its core principle is that the most effective learning occurs when there is a focus on solving real-world problems. It proposes that learning should be composed of five phases: (a) real word problem-centered, (b) activation of prior experience, (c) demonstration of skills, (d) application of skills, and (e) integration of these skills into real-world activities. This model is considered to be a high-quality and efficient approach that meets learning process requirements and the needs of learners’ psychological development [19]. In recent years, this model has been applied to medical instruction and doctor-patient communication [20, 21]. However, few studies have reported its application in patient education.

Given the previous results on the effects of hemodialysis patients’ education and the application of the theory on the First Principles of Instruction, we hypothesized that the intensive education guided by the First Principles of Instruction could help to improve hemodialysis patients’ knowledge and behavior on hyperphosphatemia, and improve the serum phosphorus control as a consequence. Thus employing the First Principles of Instruction as the frame of the program, we developed a patient education program on phosphate control and applied it to hemodialysis patients with hyperphosphatemia. The purpose of this study was to develop an intensive education program focusing on phosphate control and to explore the effectiveness on knowledge of prevention, serum phosphorus, and adherence to using phosphate binders among hemodialysis patients.

## Methods

### Design

A 6-month non-randomized, single-arm trial was used to test the effectiveness of the education program among hemodialysis patients. Data were collected before the education program and 6 months followed-up after the intervention.

### Participants and setting

We enrolled patients who were treated with maintenance hemodialysis in a hemodialysis center in a teaching hospital in Zhuhai, China, from January to June 2019. Patients were included in the education program if they were: (a) aged ≥18 years old, (b) received regular sufficient dialysis (2–3 times a week) for 3 months or longer, and (c) with pre-dialysis serum phosphorus level > 1.78 mmol/L or prescribed with oral phosphate binder (e.g., calcium carbonate, calcium acetate, lanthanum carbonate, svalam hydrochloride, etc.) to control serum phosphorus. Patients were excluded if they: (a) were hospitalized during the program, (b) had disturbance of consciousness, visual or hearing impairment, inability to communicate verbally or complicated with severe disease of heart (e.g. the New York Heart Association class IV heart failure or malignant arrhythmia), brain, lung, liver and other vital organs, and (c) had a history of total parathyroid resection/subtotal resection. Through cluster sampling, a total of 366 eligible patients were enrolled in this study, and 346 patients completed the 6-month education program.

### Implementation

This study involved an intensive education program, which was guided by The First Principles of Instruction model and was tailored, focusing on phosphate control among hemodialysis patients with hyperphosphatemia. The strategies used in the education program are described below.

#### Establishment of the phosphate control team

The phosphate control team was built by the health care professionals in the hemodialysis center at the program planning stage, which consisted of three nephrologists and eight registered nurses, and all the team members had been involved in hemodialysis for > 5 years. Two training sessions for nephrologists and nurses in the center were organized by the team, in which phosphate control was focused. During the training sessions, the nephrologists in the team introduced the KDIGO guidelines, the Chinese CKD-MBD guidelines, and related literature on diet and nutrition management of hemodialysis patients, and the First Principles of Instruction model and relevant theory were also introduced. A quiz was conducted after training, and those who passed the quiz were qualified to implement hyperphosphatemia intervention. The team was also responsible for preparing the materials such as posters, presentation slides, social media and videos, patient booklet, the patient education form, and the questionnaire assessing patients’ knowledge of phosphate control. These materials were featured phosphate control and were used for recording and evaluating during the education program. The teaching materials were updated every 3 months by the team.

#### Problem-centered approach

The First Principles of Instruction model holds that learners can effectively learn only when they become engaged in, analyze, and solve a series of practical problems in life [17]. During the implementation of this program, health care professionals delivered a patient booklet on phosphate control to the patients with hyperphosphatemia, and they took the responsibilities to guide patients to realize the importance of maintaining the serum phosphorus at a normal level and help them focus their problems on how to control serum phosphorus effectively in the daily life.

#### Activation

The model holds that the learners’ prior knowledge should be activated during instruction, and knowledge recollection can effectively promote learning [21]. During this stage, the patients with hyperphosphatemia were guided to recall and think about their existing knowledge of phosphate control. The Patient Questionnaire on Phosphate Control Knowledge was used to assess patient knowledge about hyperphosphatemia, including the definition, clinical manifestations, risks of hyperphosphatemia, and measures to control the serum phosphorus at a normal level. Informed by the questionnaire answers, the health care professionals collected the information on knowledge areas that the patients lacked in phosphate control, the most interesting questions regarding phosphate control, and the adherence to using phosphate binders. Discussion about these topics would be addressed between health professionals and patients, which aroused the attention of patients and stimulated their desire to learn the relevant knowledge.

#### Demonstration

The First Principles of Instruction model emphasizes that the demonstration of what is to be learned is more effective at promoting learning than merely imparting the relevant information [21]. Health care professionals employed multiple strategies to conduct comprehensive health education for patients with hyperphosphatemia.

First, a poster of the education program on phosphate control was designed and placed on the hemodialysis center bulletin board by the team member, and nurses notified the patients and their families about the health education program one by one to increase their enthusiasm.

Second, group-based lectures were given to patients and their family members in the waiting area of the hemodialysis center. The content of the lectures included four topics: calcium and phosphorus regulation and the formation of hyperphosphatemia, the harm of hyperphosphatemia, hemodialysis, and medication on hyperphosphatemia, and dietary management (Table 1). The presentation slides and relevant materials for the lectures were prepared by the phosphate control team. The speakers in the lectures were selected from qualified nephrologists or nurse specialists who passed the quiz after the training for phosphate control and would like to give the lecture voluntarily. One topic was given in two fixed times every week, with each lecture lasting about 30–40 min, and the topic was changed to another one next week. The lectures on the four topics were repeated in the second month.

**Table 1:** The topics of group-based lectures on hyperphosphatemia for hemodialysis patients

Topic	Knowledge
Calcium and phosphorus regulation and the formation of hyperphosphatemia	• The physiological effects of calcium and phosphorus on the human body • The role of the kidney in maintaining the calcium and phosphorus balance • Causes of hyperphosphatemia in dialysis patients • The role of hyperphosphatemia in mineral and bone disorders • Comprehensive management for hyperphosphatemia in dialysis patients
Harm of hyperphosphatemia	• The incidence of hyperphosphatemia in dialysis patients • Symptoms of hyperphosphatemia • Effects of hyperphosphatemia on bone and joints • Effects of hyperphosphatemia on the function of other organs
Hemodialysis and medication on hyperphosphatemia	• The significance of using phosphate binder in patients with hyperphosphatemia • The mechanism of phosphate binder • Major types of phosphate binders • Administration of phosphate binder • Adverse reactions to phosphate binder and medication for prevention
Dietary management on hyperphosphatemia	• Recommended phosphorus intake for dialysis patients • The advantages and disadvantages of dietary phosphorus control • How to control phosphorus intake in a reasonable diet • What are the phosphorus-rich food and low phosphorus foods • Tips for reducing phosphorus in food during cooking

Third, one-on-one bedside education (one session per month) was performed by the nurses who in charge of the patients with hyperphosphatemia. The nurses evaluated patients’ knowledge on phosphate control every month according to patients’ answers from the Patient Questionnaire on Phosphate Control Knowledge, and then identified the knowledge gaps which should be strengthened. Then with the guidance of the Patient Education Form on Phosphate Control, which included the essential points for phosphate control that patients should grasp, related Q&A questions from the education form were selected, and patients were asked accordingly during dialysis sessions. When the responses were incorrect or not given, individualized education for these points was conducted immediately. Each one-on-one bedside education session was recorded by the nurse providing the education in the patient education form, which was collected and checked by the phosphate control team every 3 months.

Fourth, bulletin board and social media were used to carry out patient education on hyperphosphatemia. Posters on phosphate control were created and placed on the bulletin board of the dialysis room. Additionally, a WeChat public account concerning relevant knowledge about phosphate control was created. The nephrologists, nurses, patients, and at least one family member per patient were invited to follow this account. Tips for phosphate control and the updated information on hyperphosphatemia and CKD-MBD were regularly pushed to the patients. Patients could ask relevant questions and get feedback from the health care professionals from this WeChat public account. Moreover, 30-min multimedia videos related to the management of hyperphosphatemia were produced and played on TV in the hemodialysis center twice a day so that patients can watch the videos during dialysis.

#### Application of skills

The First Principles of Instruction model argues that learners need to apply their knowledge or skills to solve problems in order for the intervention to be effective [21]. After the first round of group-based lectures, the patients were coached by in-charged nurses to learn the related skills to control phosphorus intake in their daily life, such as how to control the amount of phosphorus in different foods, how to prepare for low phosphorus diets during cooking, how to plan the low phosphorus recipes, and how to choose the ingredients for three meals according to their taste. The patients were also guided to learn how to take phosphate binders exactly as prescribed, including correct timing, dosage, and administration.

#### Integration of skills into real-world activities

**Fig. 1:**
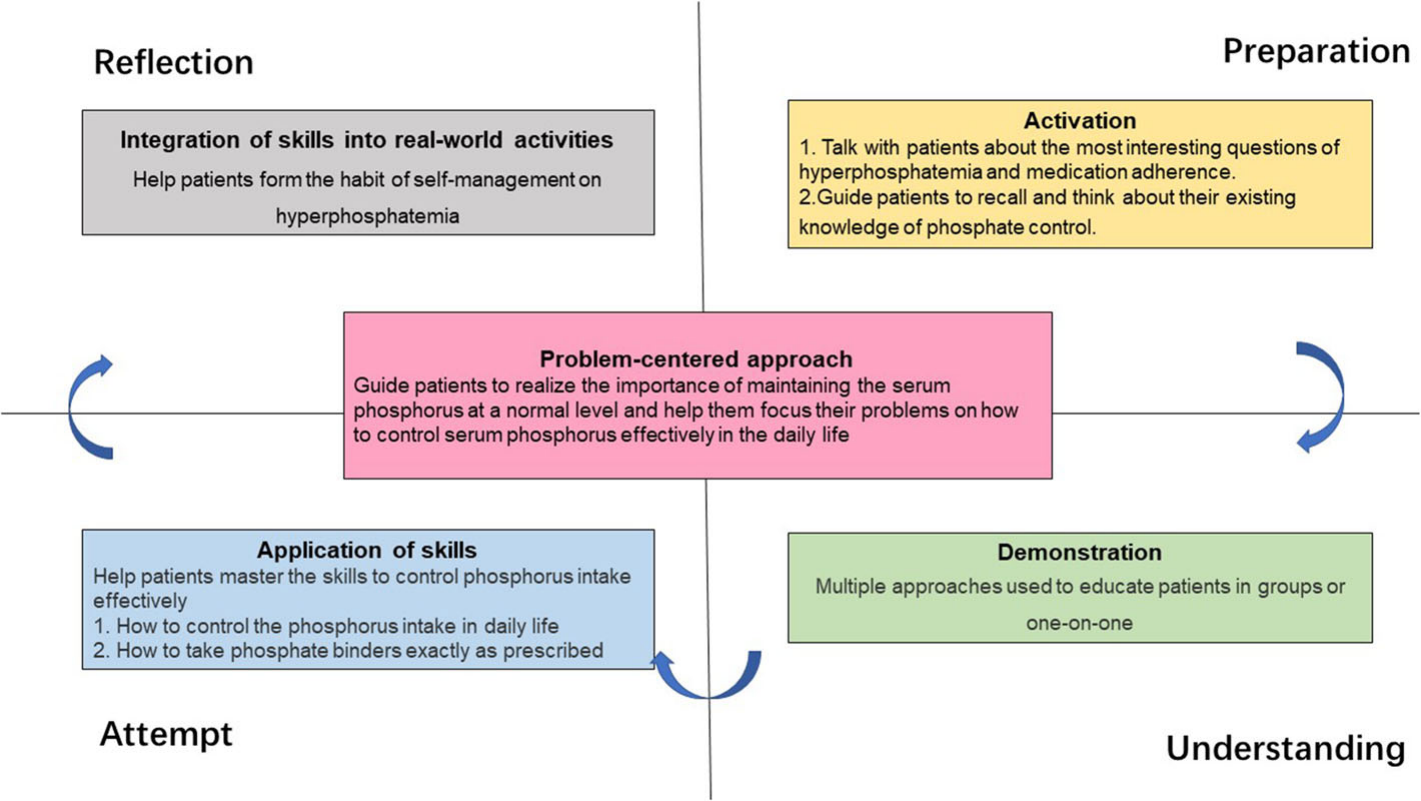
Hyperphosphatemia intensive education framework based on the First Principles of Instruction model

The First Principles of Instruction model proposes that it is necessary for learners to receive encouragement and to be able to integrate and transfer knowledge and skills into real-world activities. Experience sharing meetings were organized every 3 months for patients to enable them to communicate with each other and increase their enthusiasm for self-management. The patients who controlled serum phosphorus successfully were encouraged to share their experiences during the meeting (Fig. 1).

### Measurements

#### Sociodemographic and clinical data of the participants

We developed a questionnaire to collect the participants’ sociodemographic and clinical data. Sociodemographic data, including gender, age, educational level, were provided by the participants. Clinical data of the participants, including primary disease, dialysis frequency, dialysis mode, and pre-dialysis serum phosphorus were obtained from medical records.

#### Knowledge of phosphate control

The Patient Questionnaire on Phosphate Control Knowledge was developed by the phosphate control team by focus group discussion and two rounds of revisions. The questionnaire includes hyperphosphatemia associated knowledge, selection of low phosphorus diet and cooking skills to lowering phosphorus, and the role and administration of phosphate binders. The questionnaire consists of 25 questions, including 20 single choice questions and five multiple-choice questions with 4 points for each question, and the total score ranges from 0 to 100 points.

#### Adherence to phosphate binder

Patient’s adherence to phosphate binder was assessed by the Medication Adherence Report Scale (MARS), which consists of five items describing non-adherent behavior [22]. Each item is scored with 1 (‘always’) to 5 points (‘never’), leading to a sum score ranging between 5 and 25 points. Adding up all items yields a total score with a score equal to or greater than 24 suggesting medication adherence [23]. The scale showed higher test-retest reliability (*r* = 0.987, *P*<0.001).

### Data collection

The nurses in the phosphate control team distributed the medication adherence report scale to the participants before and 6 months after the program. Patients who participated in the study completed the scale during dialysis, and the nurses would help them fill out the scale if needed. The data of knowledge phosphate control before and after the 6-month program were collected by in charged nurses and downloaded by the phosphate control team from the online survey tool Wenjuanxing (https://www.wjx.cn/) and the sociodemographic and disease characteristics were collected from the patients’ medical records.

### Statistical analysis

SPSS 21.0 software was used to analyze the data. The descriptive statistics were used to describe the patients’ characteristics, serum phosphorus level, knowledge on phosphate control, and adherence to phosphate binder, in which mean and standard deviation were used to describe the continuous data, and frequency and percentage were used to describe the categorical data. The *chi-square* test was used to analyze the changes of the controlled percentage of serum phosphorus and the percentage of adherence to phosphate binder after the program. The paired sample *T* test was used to compare the changes of the patients’ scores on knowledge of phosphate control and the changes of scores on adherence to phosphate binder after the program. *P* < 0.05 indicated statistical significance.

### Ethics of the study

This study was approved by the ethics committee of the fifth affiliated hospital of Sun Yat-sen University (approval number: K71–1). All the patients who participated in the study had fully informed the rights in the study in accordance with the Declaration of Helsinki and had signed an informed consent form before participated in the study.

## Results

### Characteristics of participants

Among 366 patients participated in this study, 224 (61.2%) were male and 142 (38.8%) were female. They were aged 18–88 years (mean: 41.2 ± 5.2 years) with hemodialysis duration for 0.5–20 years. There were 71 patients (19.4%) with primary school or below, 220 patients (60.1%) with middle school, and 75 patients (20.5%) with the college or above. Most of the patients (320, 87.4%) received dialysis 3 times per week. One hundred thirteen patients (30.9%) had hemodialysis (HD) only, and 253 (69.1%) had HD+ hemodiafiltration(HDF) (patients received hemodiafiltration once or twice a week in addition to hemodialysis once or twice a week). The primary diseases were as follows: chronic glomerulonephritis (54.9%), diabetic nephropathy (19.7%), hypertensive nephropathy (7.4%), polycystic kidney disease (7.1%), obstructive nephritis (3.8%), immune nephropathy (3.8%), and other diseases (3.3%). Patients used lanthanum carbonate (44.5%), calcium acetate (39.1%), calcium carbonate (31.1%), and/or sevelamer carbonate (15.1%) to control hyperphosphatemia, while 9.4% of them without phosphate binder. The average levels of hemoglobin, serum albumin, calcium, phosphorus and Kt/V were 105.2 ± 17.3 g/L, 38.6 ± 3.2 g/L, 2.1 ± 0.2 mmol/L, 2.0 ± 0.6 mmol/L and 1.4 ± 0.3, respectively (Table 2). The serum levels of albumin increased compared with before intervention (*t* = − 6.073, *P*<0.05), and there was no significant changes in body weight (t = − 1.386, *P*>0.05) and serum creatinine (*t* = − 1.797, *P*>0.05) after the program. For the experience sharing meetings, 80% of the patients participated once and 50% of the patients participated twice. Twenty patients dropped out of the program during the 6-month follow up, including 9 of them were transferred to other hospitals, 5 were hospitalized, 2 were transferred to peritoneal dialysis, 1 received kidney transplant, and 2 died. There were no significant differences between the dropped out patients and patients who complicated the program on the sociodemographic and clinical characteristics.

**Table 2:** Sociodemographic and clinical data of the participants

Category		N(%)	Mean ± SD
Gender	Male	224 (61.2%)	
	Female	142 (38.8%)	
Age(year)			41.2 ± 5.2
Education level	Primary school or below	71(19.4%)	
	Middle school	220 (60.1%)	
	College or above	75(20.5%)	
Dialysis frequency	3 times / week	320 (87.4%)	
	2 times / week	46(12.6%)	
Dialysis mode	HD	113(30.9%)	
	HD + HDF	253 (69.1%)	
Blood tests	Hemoglobin (g/L)		105.2 ± 17.3
	Serum albumin(g/L)		38.6 ± 3.2
	Calcium(mmol/L)		2.1 ± 0.2
	Phosphorus(mmol/L)		2.0 ± 0.6
	Serum creatinine(μmol/L)		1081.8 ± 16.2
	Kt/ V		1.4 ± 0.3
Dry weight(kg)			59.1 ± 12.4
The primary diseases	Chronic glomerulonephritis	201(54.9%)	
	Dabetic nephropathy	72(19.7%)	
	Hypertensive nephropathy	27(7.4%)	
	Polycystic kidney disease	26(7.1%)	
	Obstructive nephritis	14(3.8%)	
	Immune nephropathy	14(3.8%)	
	Other diseases	12(3.3%)	
Phosphate binder	Lanthanum carbonate	163(44.5%)	
	Calcium acetate	143(39.1%)	
	Calcium carbonate	114(31.1%)	
	Sevelamer carbonate	55(15.1%)	

*Abbreviations*: *HD* hemodialysis, *HDF* hemodiafiltration

### Controlled percentage of serum phosphorus before and after the program

The proportion of controlled serum phosphorus significantly increased from 43.5 to 54.9% after the program (*Chi-square* = 23,808, *P*<0.001, Table 3).

**Table 3:** The proportion of controlled serum phosphorus before and after the program

	≤1.78 mmol/L (%)	> 1.78 mmol/L (%)	*Chi-square*	*P*
Before the program	146 (43.5)	220 (56.5)	23.808	< 0.001
6 months after program	201 (54.9)	145 (45.1)		

### The knowledge of phosphate control before and after the program

The average score on patients’ knowledge of phosphate control before the program was 59.0 ± 18.9, and it increased to 80.6 ± 12.4 after the program (*t* = −17.789, *P*<0.001). The average scores on knowledge of hyperphosphatemia, knowledge of diet and knowledge of phosphate binders were increased significantly after the program (*P*<0.001, Table 4).

**Table 4:** Comparison of the knowledge of phosphate control before and after the program (*N* = 346)

Time	Total score	Knowledge of disease	Knowledge of diet	Knowledge of phosphorus binder
Before program	59.0 ± 18.9	25.0 ± 10.2	26.7 ± 8.4	7.3 ± 4.1
6 months after program	80.6 ± 12.4	34.1 ± 8.2	36.5 ± 4.4	10.1 ± 2.5
*t*	−17.789	−12.864	−19.077	−11.024
*P*	< 0.001	< 0.001	< 0.001	< 0.001

### Adherence to phosphate binders before and after the program

The average score of patients’ adherence to phosphate binders before the program was 18.8 ± 3.7, and it significantly improved to 22.5 ± 2.9 (*t* = −20.245, *P*<0.001) after the program. The percentage of adherence to phosphate binders significantly increased from 21.9% before the program to 44.5% after the program (*Chi-square* = 42.239, *P*<0.001, Table 5).

**Table 5:** The proportion of adherence to phosphate binder before and after the program

	Adherence (%)	Non-adherence (%)	*Chi-square*	*P*
Before program	80 (21.9)	286 (78.1)	42.239	< 0.001
6 months after program	162 (44.5)	202 (55.5)		

## Discussion

Although the First Principles of Instruction model has been widely used in school education and adult education [24–26], few studies in China have applied it to patient education. This study is the first time to apply this model to the patients’ education on phosphate control among hemodialysis patients in China, and it showed that the controlled percentage of serum phosphorus, patients’ knowledge and adherence to phosphate binder could be improved dramatically.

This study showed that after the intensive education focusing on phosphate control was implemented, the knowledge of phosphate control among hemodialysis patients with hyperphosphatemia was significantly improved compared with that before the program (59.0 ± 18.9 vs. 80.6 ± 12.4, *P* < 0.001). According to the theory of Knowledge, Attitude, Belief, and Practice (KABP), behavior change can be divided into three processes: acquiring knowledge, generating belief and forming behavior [27]. The conversion of knowledge into behavior requires external factors, with health education being an important external factor. Patients’ knowledge of phosphate control learned through health education is the prerequisite to gradually form a reasonable and sustainable adherence behavior to phosphate binder and low phosphorus diet. Integrating with appropriate teaching strategies during patient education, nurses can effectively promote the transformation of the knowledge into corresponding behavior [28]. The results of this study suggest that the intensive education program, guided by the First Principles of Instruction model, can effectively improve the knowledge of phosphate control among dialysis patients with hyperphosphatemia.

Mahler [23] used the MARS to investigate the medication compliance of patients with cardiovascular diseases and the results showed that 77% of patients had high compliance, while Ohya [29] used other tools to investigate the medication compliance of phosphate binders in hemodialysis patients. The results showed that 76.7% of patients had high compliance in the investigation by Wileman [30] on the compliance of 112 hemodialysis patients with phosphorus binders and the mean MARS score was 21.2 (SD = 4.5). In this study, the mean MARS score of patients increased significantly from 18.8 ± 3.7 to 22.5 ± 2.9 after the intervention. A high level of medication adherence requires a high degree of awareness, knowledge, and skills related to phosphate control. The transformation of knowledge into behavior depends on comprehensive phosphate control guidance by health care professionals [31]. In this study, the low adherence of patients to phosphate binder before the program may be related to the patients’ low level with phosphate control-related knowledge, including the harm of hyperphosphatemia, the selection of low-phosphorus foods and the use of phosphate binder. Patients’ adherence to phosphate binder after the intensive education program was significantly improved (*P* < 0.001). Xiong et al. [32] found that the education program employed the First Principles of Instruction model could help to improve the knowledge of prevention and treatment of hyperkalemia among the family members of hemodialysis patients. Thus, the results showed that the intensive education program applied with the First Principles of Instruction model and tailored for the hemodialysis patients with hyperphosphatemia was effective for the improvement of adherence to phosphate binder.

The proportion of controlled serum phosphorus significantly increased from 43.5% before the program to 54.9% after the program (*Chi-square* = 23,808, *P <* 0.001), indicating the serum phosphorus of patients was effectively controlled. The knowledge on phosphate control and adherence to phosphate binder are important factors affecting the serum phosphorus level. Followed with the improvement of the knowledge, skills, and adherence behavior related to phosphate control during education, as the most important outcome, more patients could maintain the serum phosphorus at a normal level as a consequence.

Different from the traditional instructional models, the First Principles of Instruction model is featured for problem-solving during education. It is carried out in five stages, which are continuously repeated in a cycle. It values the diversity of instructional methods, putting instructional theory into practice, and continuity of the instructional process. Serum phosphorus control is crucial for hemodialysis patients with hyperphosphatemia, and it must be addressed. Efforts should be made to increase patients’ attention to the risks of hyperphosphatemia and ensure that they actively participate in the practice on how to control the serum phosphorus level. This is followed by a gradual activation of the patients’ prior knowledge, identification of knowledge gaps, and elicitation of their curiosity.

Moreover, through monthly assessments, attempts were made to assess the effect of education, check for omissions and compensate for them, and form a cycle to ensure the continuity, high-quality of education, and ultimately to improve the knowledge and skills of patients and family members. After the group-based lectures and one-on-one education, patients’ knowledge and skills related to phosphate control were strengthened, and patients had known how to choose low phosphorus foods, prepare low phosphorus recipes, and describe cooking skills and the use of phosphate binder. This instruction model help patients not only remember the knowledge but also effectively put it into practice, so that converted the abstract concepts into specific practices.

Another important factor facilitating the efficacy of the intentive education program was multiple strategies employed to disseminate information for patients, such as putting up posters, organizing group-based lectures, intensive one-on-one education, use of the video, and use of WeChat platform. These approaches increased physician/nurse-patient interactions and maintained the patients’ enthusiasm to learn new knowledge. Additionally, patients exchanged their experiences in the experience sharing meeting, which not only promoted them to transform the knowledge they had learned into practice but also encouraged them to share useful knowledge and skills so that they could learn from each other. In this way, patients were empowered to manage their health in daily life.

## Limitations

Although this is the first study that explored the effectiveness of the intensive education program guided by the First Principles of Instruction model among hemodialysis patients on phosphate control, this study existed some limitations. Firstly, the patients before the program were regarded as the control group, so the participants were not randomized, and secondly, blinding was not used in the study. Since the non-randomized, single-arm trial was designed in this study, the potential confounding factors on the results could not be effectively controlled. Meanwhile, the outcomes in this study would be affected by some biases such as Hawthorn’s effect, so the effectiveness of this program should be taken with caution. The main reason why this study did not use randomized grouping and blinding was that many approaches in interventions need to be performed in the dialysis center in public, such as publicity column, TV video, and the program required all the health care professionals involving in the program. Therefore all the patients and health care professionals in this hemodialysis center would inevitably receive the intervention. Since the randomized controlled trial is the gold standard in clinical studies, the multicenter, cluster-randomized parallel control design will help to test the effectiveness of this intervention model in-depth in the future.

## Conclusion

The intensive education program on phosphate control for hemodialysis patients with hyperphosphatemia can effectively increase the controlled percentage of serum phosphorus, improve patients’ knowledge of phosphate control, and promote patients’ adherence to phosphate binders.

## Data Availability

The datasets used and/or analyzed during the current study are available from the corresponding author on reasonable request.

## Abbreviations

MBD: Mineral and bone disorder
CKD: Chronic kidney disease
DOPPS: Dialysis Outcomes and Practice Pattern Study
KDOQI: Kidney Disease Outcomes Quality Initiative
KDIGO: The Kidney Disease Improving Global Outcomes
CKD-MBD: Chronic Kidney Disease-Mineral and Bone Disorder
MARS: Medication Adherence Report Scale
HD: Hemodialysis
HDF: Hemodiafiltration
